# The Role of Pretherapy Quantitative Imaging and Dosimetry in Radioiodine Therapy for Advanced Thyroid Cancer

**DOI:** 10.2967/jnumed.122.264913

**Published:** 2023-07

**Authors:** Jan Taprogge, Carla Abreu, Siraj Yusuf, Gemma Ainsworth, Rachel H. Phillip, Jonathan I. Gear, Rebecca Gregory, Francesca Leek, Iain Murray, Amy B. Coulson, Sarah R. Brown, Yong Du, Kate Newbold, Jonathan Wadsley, Glenn D. Flux

**Affiliations:** 1 Joint Department of Physics, Royal Marsden NHSFT, Sutton, United Kingdom;; 2 Institute of Cancer Research, London, United Kingdom;; 3 Department of Nuclear Medicine and PET/CT, Royal Marsden NHSFT, Sutton, United Kingdom;; 4 Clinical Trials Research Unit, Leeds Institute of Clinical Trials Research, University of Leeds, Leeds, United Kingdom;; 5 Thyroid Unit, Royal Marsden NHSFT, Sutton, United Kingdom; and; 6 Department of Oncology, Weston Park Hospital, Sheffield, United Kingdom

**Keywords:** dosimetry, radioiodine, theragnostics, advanced thyroid cancer

## Abstract

Radioactive iodine is well established as a successful treatment for differentiated thyroid cancer (DTC), although around 15% of patients have local recurrence or develop distant metastases and may become refractory to radioactive iodine (RAI). A personalized approach to treatment, based on the absorbed radiation doses delivered and using treatments to enhance RAI uptake, has not yet been developed. **Methods:** We performed a multicenter clinical trial to investigate the role of selumetinib, which modulates the expression of the sodium iodide symporter, and hence iodine uptake, in the treatment of RAI-refractory DTC. The iodine uptake before and after selumetinib was quantified to assess the effect of selumetinib. The range of absorbed doses delivered to metastatic disease was calculated from pre- and posttherapy imaging, and the predictive accuracy of a theranostic approach to enable personalized treatment planning was investigated. **Results:** Significant inter- and intrapatient variability was observed with respect to the uptake of RAI and the effect of selumetinib. The absorbed doses delivered to metastatic lesions ranged from less than 1 Gy to 1,170 Gy. A strong positive correlation was found between the absorbed doses predicted from pretherapy imaging and those measured after therapy (*r* = 0.93, *P* < 0.001). **Conclusion:** The variation in outcomes from RAI therapy of DTC may be explained, among other factors, by the range of absorbed doses delivered. The ability to assess the effect of treatments that modulate RAI uptake, and to estimate the absorbed doses at therapy, introduces the potential for patient stratification using a theranostic approach. Patient-specific absorbed dose planning might be the key to more successful treatment of advanced DTC.

## Visual Abstract

**Figure:**
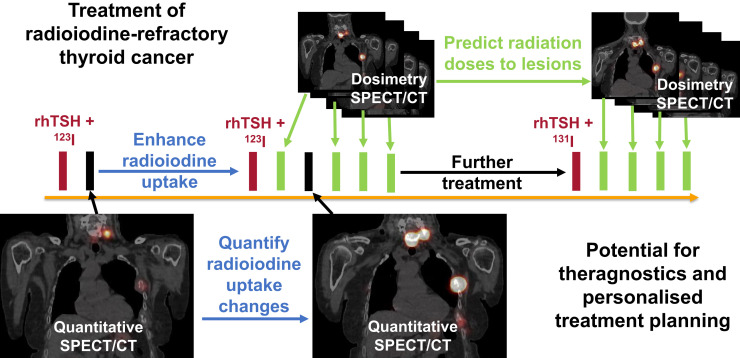

Differentiated thyroid cancer (DTC) has been treated with radioactive iodine (RAI) for over 80 y (1). More than 580,000 new DTC cases were estimated worldwide for 2020 (2). Although 84% of patients survive for 10 y or more (3), over 15% of patients have local recurrence or develop distant metastases, and 5%–10% eventually become RAI-refractory (4*,*^5^). This status carries a poor prognosis with a median overall survival of 3–5 y. Treatment with lithium carbonate, retinoic acid, or histone deacetylase inhibitors has been attempted to resensitize disease to RAI treatment, but no significant clinical benefit has been demonstrated (3*,*^4^).

Initial results have shown that mitogen-activated protein kinase/extracellular-signal–regulated kinase pathway inhibitors such as the mitogen-activated protein kinase inhibitor selumetinib (ARRY-1428860) might be used to increase sodium iodide symporter expression and restore or enhance uptake of RAI (6*–*8). Further benefit may be gained from a theranostic approach (9), which offers the possibility of personalized treatments by combining therapeutics and diagnostics. In the case of RAI for DTC, the widely available imaging isotope ^123^I-NaI may be used to guide treatment with ^131^I-NaI and to predict the absorbed doses delivered to lesions and to healthy organs (10*,*^11^).

Here, we report the imaging and dosimetry results from a phase 2 trial (SEL-I-METRY, EudraCT no. 2015-002269-47) to resensitize RAI-refractory DTC to further RAI therapy (12*,*^13^). The aims of this aspect of the trial were to establish the quantitative increase in RAI uptake due to selumetinib and the range of absorbed doses delivered to metastatic disease from fixed levels of administered activity. In addition, we aimed to determine the accuracy with which the absorbed doses delivered at therapy may be predicted from pretherapy diagnostic studies and to establish the percentage of lesions responding to treatment.

## MATERIALS AND METHODS

### Study Design

Patient inclusion and exclusion criteria are provided in Supplemental Table 1 (supplemental materials are available at http://jnm.snmjournals.org). Iodine-refractory disease was defined as one or more lesions with no measurable iodine uptake or an iodine-avid lesion that progressed within 12 mo of RAI. An exploratory endpoint of the SEL-I-METRY trial was to assess the feasibility of quantitative imaging and SPECT/CT-based lesion dosimetry to personalize treatment for patients with advanced DTC.

**FIGURE 1.**
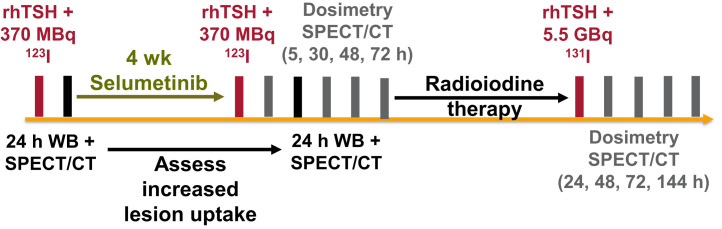
SEL-I-METRY imaging schedule consisting of 24-h whole-body and SPECT ^123^I-NaI scans before selumetinib, 24-h whole-body and SPECT ^123^I-NaI scans after selumetinib, up to 4 additional dosimetry SPECT/CT ^123^I-NaI scans after selumetinib, and up to 4 dosimetry SPECT/CT scans after treatment with 5.5 GBq of ^131^I-NaI. rhTSH = recombinant human thyroid-stimulating hormone; WB = whole body.

Participants received 75 mg of selumetinib orally twice daily for 4 wk (Fig. 1). Pre- and postselumetinib quantitative ^123^I-NaI SPECT/CT and whole-body scans were used to predict the increase in ^131^I-NaI uptake for subsequent treatment after the initial 4 wk of selumetinib. Patients with an increase in ^123^I-NaI uptake of more than 30% after selumetinib in at least one lesion went on to receive RAI therapy with a fixed activity of 5.5 GBq of ^131^I-NaI (13). Patients continued receiving selumetinib until the ^131^I-NaI therapy (maximum, 18 d) during the ^123^I-NaI scan review.

For patients eligible for therapy, ^123^I-NaI dosimetry was performed after the 4 wk of selumetinib administration (referred to here as pretherapy dosimetry) and lesional dosimetry was performed after ^131^I-NaI therapy (referred to here as posttherapy dosimetry). Pretherapy dosimetry consisted of up to 5 SPECT/CT scans (at 5, 24, 30, 48, and 72 h) after administration of 370 MBq of ^123^I-NaI. After ^131^I-NaI therapy, SPECT/CT was performed at 24, 48, 72, and 144 h (Fig. 1). Recombinant human thyroid-stimulating hormone stimulation was administered before ^123^I-NaI and ^131^I-NaI. The imaging and reconstruction protocols are provided in Supplemental Tables 2 and 3.

The study was approved by East Midlands, the Leicester South Research Ethics Committee (15/EM/0455), the institutional review boards of participating centers, and the Medicines and Health Care Products Regulatory Agency. All patients provided written informed consent before trial registration.

### Quantitative Imaging to Assess Effect of Selumetinib

γ-cameras at participating centers were configured for quantitative imaging including the determination of calibration factors for ^123^I-NaI and ^131^I-NaI and dead-time correction factors for ^131^I-NaI (14). Anatomic lesion volumes were outlined by a trained radiologist on each of the CT components of up to 5 postselumetinib ^123^I-NaI SPECT/CT scans. Lesions were excluded from the analysis if the largest diameter was smaller than 10 mm. Oversized volumes of interest, encompassing all activity visible within the lesions, were delineated to determine activity retention in all ^123^I-NaI and ^131^I-NaI SPECT/CT images. This approach was used to minimize problems arising from breathing motion and partial-volume effects and, therefore, allows for dosimetry estimates of small lesions.

The effect of selumetinib was assessed by calculating the absolute and relative differences in ^131^I-NaI lesion uptake during therapy, as predicted from the pre- and postselumetinib ^123^I-NaI images. These were converted to predictions of ^131^I-NaI uptake during therapy, taking into account the differences in physical half-lives and administered activities.

### Predictive Accuracy of Pretherapy Dosimetry and Treatment Planning

Pretherapy dosimetry was performed to predict lesional absorbed doses during ^131^I-NaI therapy, taking into account the differences in physical half-life and injected activities, to investigate the potential of personalized treatment planning in this cohort. Dosimetry was performed according to the MIRD formalism (15) using mass-adjusted S values and taking into account only self-dose (16). Uncertainties regarding absorbed doses were estimated according to the guidance of the European Association of Nuclear Medicine (17).

The predictive accuracy of the pretherapy dosimetry was assessed by calculating the absolute and relative differences between the absorbed doses predicted before therapy and those measured after therapy.

### Lesion Response Assessment

Response after RAI treatment was assessed using RECIST (18). The analysis was performed on a lesion-by-lesion basis between the baseline CT scan and the latest follow-up CT scan (maximum, 12 mo) after RAI treatment. Complete response (CR) was defined as lesion disappearance. Partial response (PR) was a decrease in lesion size by at least 30% (longest axis, in mm). Progressive disease was an increase in lesion size by at least 20% (longest axis, in mm). Stable disease was a case with no CR, PR, or progressive disease. Overall response was observation of either CR or PR, whereas clinical benefit was achievement of CR, PR, or stable disease.

### Statistical Analysis

The Kruskal–Wallis test was used to assess whether relative changes in quantitative lesional uptake before and after selumetinib treatment significantly differed between patients. The relationship between relative changes in quantitative uptake before and after selumetinib treatment and baseline uptake was assessed using the Spearman rank correlation coefficient. The relationship between absorbed doses from pretherapy and posttherapy dosimetry was assessed using Pearson product-moment correlation coefficients. To account for the possibility of multiple, nonindependent lesions within a single patient, all correlation coefficients were calculated on group-mean–centered data.

The relationships between postselumetinib uptake and absorbed doses, and among the baseline data, biomarker, and selumetinib treatment parameters, were explored. A multilevel modeling approach was used to account for the nested data structure, incorporating random effects with respect to each patient. Lesional variables (baseline CT lesion size) were explored as fixed effects within the model. Patient-level variables (cancer subtype, sum of CT lesion size, thyroglobulin, and total selumetinib dose) were explored as random effects in the model. All models were adjusted for preselumetinib lesion uptake or pretherapy predicted absorbed doses, respectively, as a fixed effect. A forward selection approach with χ^2^ testing was used to decide which variables to include in the model. χ^2^ tests were used to test the difference between the nested models and determine whether inclusion of a variable improved the model fit.

The association between treatment success and quantitative absorbed doses from pre- and postselumetinib ^123^I-NaI and ^131^I-NaI imaging was investigated using univariate logistic regression modeling. Additionally, receiver-operating-curve analysis of the data was explored to establish a threshold absorbed dose for overall response rate and clinical benefit rate, using cutoffs at the median, one-third and two-thirds quantiles.

All statistical tests were exploratory because the trial was not formally powered to detect statistically significant effects on the dosimetry endpoints. Testing was performed at the 2-sided 5% significance level and did not account for multiple testing. Statistical analysis was performed using R, version 4.0.2 or later versions, and the Kruskal–Wallis test was performed using GraphPad Prism, version 9.3.1 (GraphPad Software), for Microsoft Windows.

## RESULTS

**FIGURE 2.**
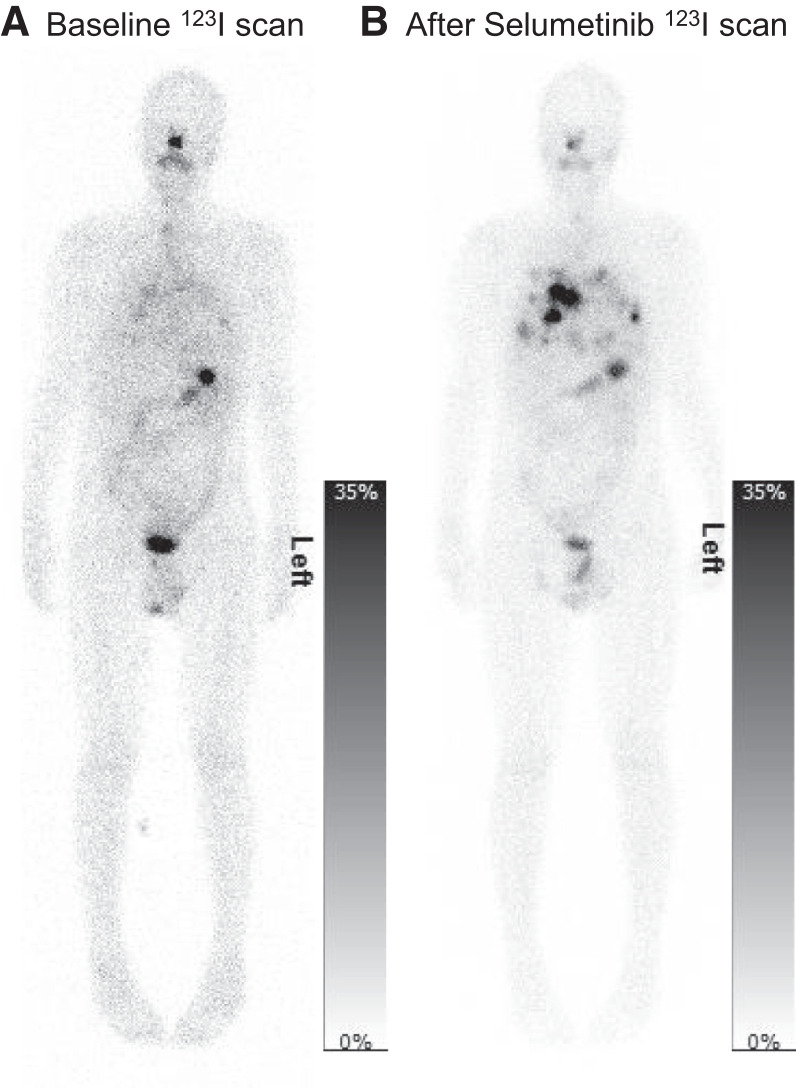
Example of whole-body planar scans at 24 h after ^123^I-NaI administration at baseline, before selumetinib was administered (A), and after 4 wk of treatment with selumetinib (B).

Thirty RAI-refractory DTC patients were recruited to SEL-I-METRY, of whom 28 received selumetinib treatment. After 4 wk of selumetinib, 9 patients (Table 1) demonstrated an increase in ^123^I-NaI uptake of 30% or more (Fig. 2) and were administered RAI treatment after a median of 12.5 d (range, 2–15 d). During this time, the patients continued to take selumetinib. Within these 9 patients, 39 lesions were identified. Median lesion volume was 2.6 cm^3^ (minimum, 0.3 cm^3^; 25th percentile, 0.7 cm^3^; 75th percentile, 9.6 cm^3^; maximum, 43.1 cm^3^). Eighteen lesions were in the lungs, 14 in bone, and 7 in soft tissues (4 in lymph nodes and 3 in the area of the thyroid bed or the neck).

**TABLE 1.** Patient Characteristics

Characteristic	Value
Age (y)	48 (45–78)
Female	3 (33%)
Histology subtype	
Papillary	2 (22%)
Follicular	7 (78%)
Cumulated activity of RAI before study registration (GBq)	7.6 (3.7–14.6)
Baseline thyroglobulin (μg/L)	742 (36–7,530)

Qualitative data are number and percentage; continuous data are median and range.

### Quantitative Imaging to Assess Effect of Selumetinib

**FIGURE 3.**
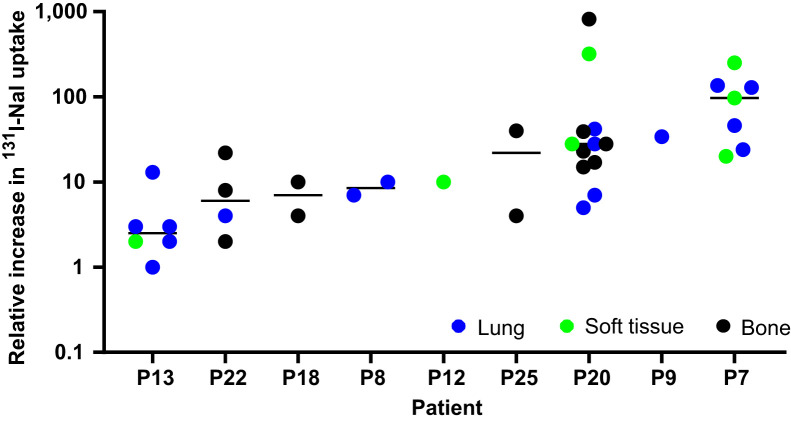
Relative change in predicted ^131^I-NaI uptake after treatment with selumetinib with respect to uptake before selumetinib administration. Relative increase in uptake is shown for 9 patients who progressed to RAI therapy. P = patient.

**FIGURE 4.**
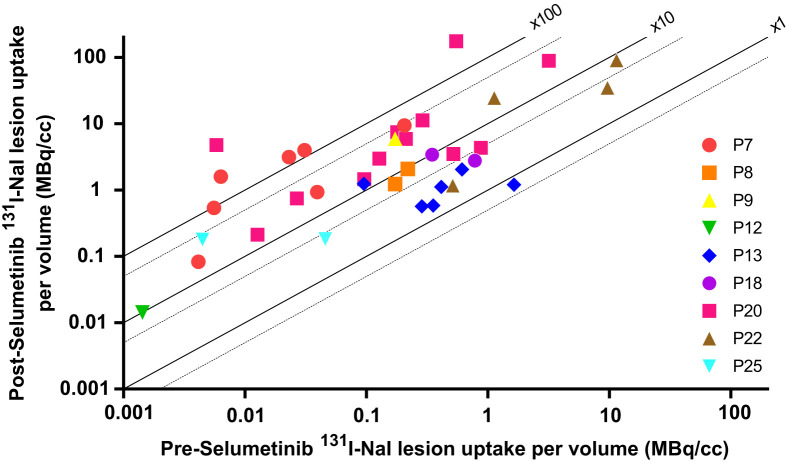
Uptake of ^131^I-NaI before and after treatment with selumetinib. Solid lines represent relative increase in uptake by factors of 1, 10, and 100, respectively, to illustrate relative effect of selumetinib in individual patients and lesions. P = patient.

**FIGURE 5.**
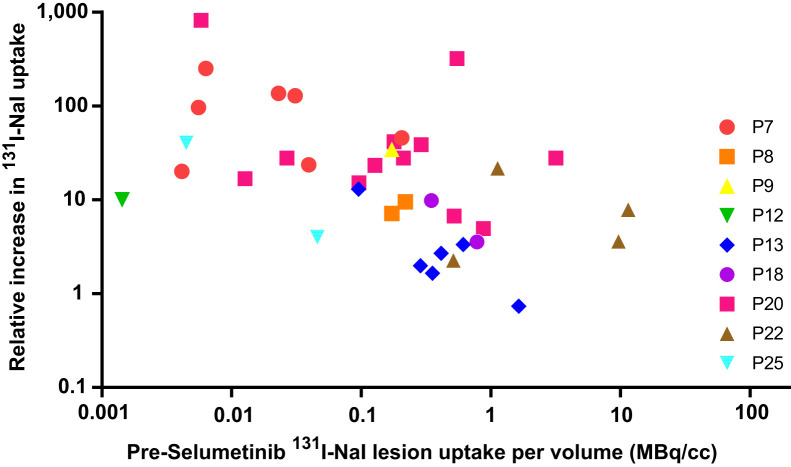
Relative increase in ^131^I-NaI uptake after treatment, with selumetinib plotted against baseline uptake of ^131^I-NaI before treatment. P = patient.

Quantitative SPECT before and after treatment with selumetinib was used to assess the effect on RAI uptake. Two lesions were excluded from this analysis because they were not included in the range of the preselumetinib scan. The median predicted ^131^I-NaI uptake per lesion volume (MBq/cm^3^) at 24 h in 37 lesions before and after selumetinib was 0.2 MBq/cm^3^ (range, 0.001–11.5 MBq/cm^3^) and 2.1 MBq (range, 0.01–175.4 MBq/cm^3^), respectively. The median absolute change (predicted uptake after selumetinib minus predicted uptake before selumetinib) and the median relative change (predicted uptake after selumetinib divided by uptake before selumetinib) were 1.9 MBq/cm^3^ (range, −0.4 to 174.9 MBq/cm^3^) and 16.7 (range, 0.7–819.1), respectively. Figure 3 shows the relative change in predicted ^131^I-NaI uptake. The absolute and relative changes in uptake are presented in Figures 4 and 5, respectively, with respect to the baseline uptake.

A large inter- and intrapatient variability was observed for the relative change in uptake on a lesional basis. A Kruskal–Wallis test showed that the relative increase in uptake significantly differed between patients (*H*_(8)_ = 22.48, *P* = 0.004). There was a weak, positive correlation between the group-mean–centered data of the relative uptake change and the baseline uptake before selumetinib (Supplemental Fig. 1), *r*_(37)_ = 0.011; however, the relationship was not significant (*P* = 0.946).

A multilevel modeling approach was used to assess the relationships between postselumetinib uptake, adjusted for preselumetinib lesion uptake, and baseline data, biomarkers, and selumetinib treatment parameters. No variables of interest improved the multilevel model fit over the null model according to the χ^2^ difference tests, with many models demonstrating singular fit, likely caused by the small sample size’s being unable to support the complexity of the modeling approach. The intraclass correlation coefficient was calculated as 0.093 for patients, indicating large variability.

### Predictive Accuracy of Pretherapy Dosimetry

To assess the feasibility of applying theranostic and dosimetry-based treatment planning in this patient cohort, pre- and posttherapy dosimetry was performed to assess the absorbed doses delivered to the lesions and to investigate whether pretherapy imaging can be used for treatment planning. All 39 lesions were evaluated for dosimetry. The median predicted absorbed doses from pretherapy and posttherapy dosimetry were 17.2 Gy (range, 0.1–1,292.1 Gy) and 10.4 Gy (range, 0.3–1,169.9 Gy), respectively. The median relative percentage difference between pretherapy and posttherapy dosimetry was −33% (range, −98% to 764%).

**FIGURE 6.**
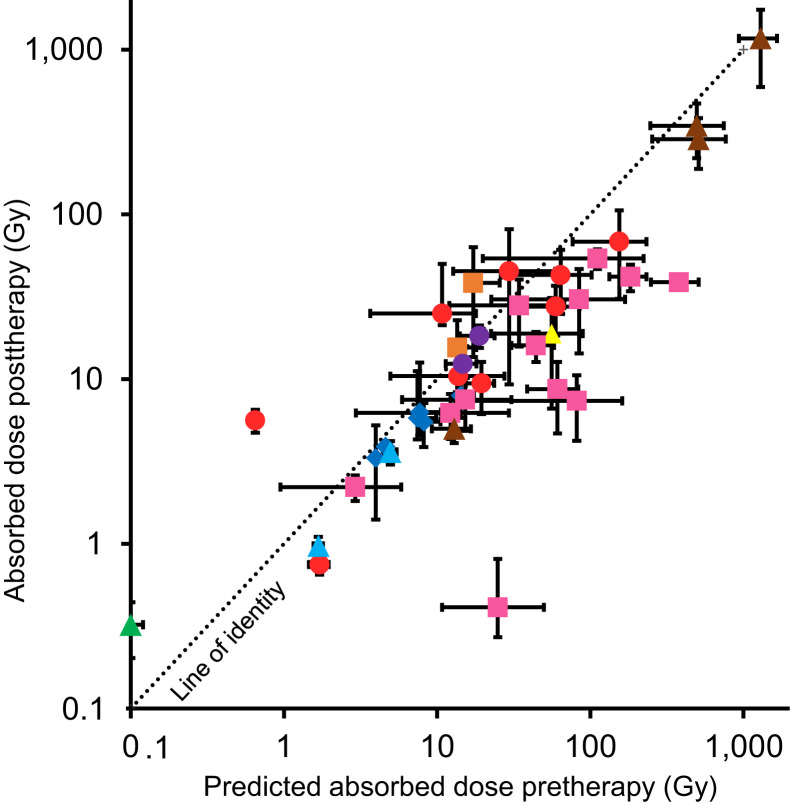
Comparison of absorbed doses for each lesion as predicted from pretherapy dosimetry and measured from posttherapy dosimetry.

**FIGURE 7.**
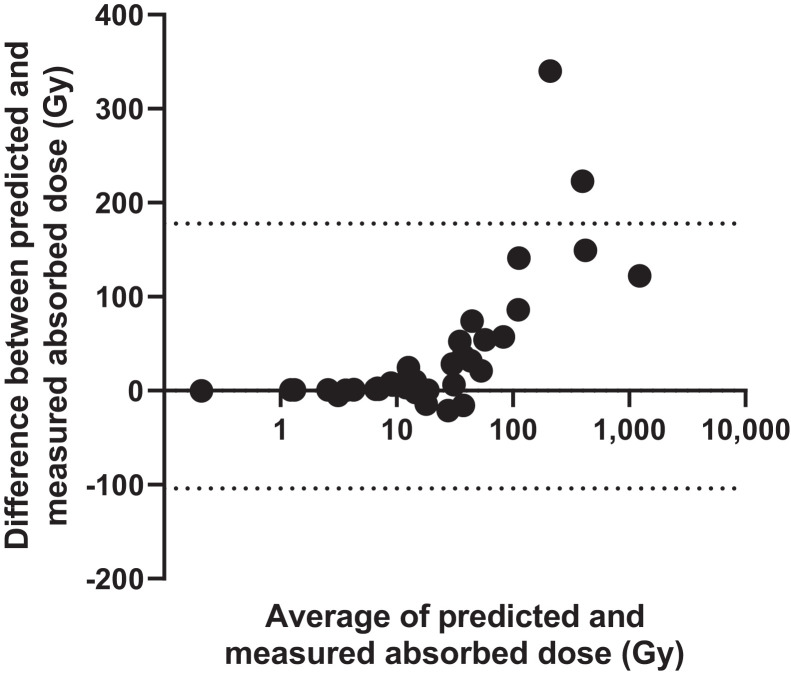
Bland–Altman plot for comparison of absorbed doses predicted and measured for each lesion, showing difference of predicted absorbed doses minus actual delivered absorbed doses.

Comparison of predicted absorbed doses before RAI therapy and measured absorbed doses after RAI therapy are shown in Figure 6, illustrating the wide range of absorbed doses delivered. Pearson product-moment correlation analysis of the group-mean–centered data (Supplemental Fig. 2) resulted in a strong positive correlation between the predicted absorbed doses from pretherapy dosimetry and posttherapy dosimetry, *r* (37) = 0.93, *P* < 0.001. Figure 7 shows a Bland–Altman plot of the difference between predicted and measured absorbed doses. The estimated bias was 37.0 Gy (SD, 71.9 Gy), illustrating that predicted absorbed doses were higher than delivered absorbed doses. Supplemental Figures 3 and 4 show Bland–Altman plots of the 24-h uptake and the retention half-lives, respectively, comparing the predicted values using pretherapy dosimetry and the values measured after therapy.

A multilevel modeling approach was used to assess the relationships between posttherapy absorbed doses, adjusted for pretherapy predicted absorbed doses, and baseline data, biomarkers, and selumetinib treatment parameters. No variables of interest improved the multilevel model fit over the null model according to the χ^2^ difference tests, again with many models demonstrating singular fit. The intraclass correlation coefficient was calculated as 0.062 for patients.

### Lesion Response Analysis

Follow-up CT scans were collected for 7 patients (last follow-up: at 3 mo in 1 patient, 6 mo in 1 patient, 9 mo in 1 patient, and 12 mo in 4 patients), with a total of 24 lesions included in the analysis. Response was assessed using RECIST (18) but on an individual-lesion basis by comparing the baseline CT scan with the latest follow-up scan for each patient. Overall response was defined as either CR (disappearance of the lesion) or PR (≥30% decrease in lesion size as defined by the longest axis, in mm). Clinical benefit was taken to be CR, PR, or stable disease (<30% decrease or increase in lesion size). Overall response and clinical benefit were observed in 13% (3/24) and 83% (20/24) of lesions, respectively.

Univariate logistic regression modeling was used to assess the association between quantitative absorbed doses from pre- and postselumetinib therapy imaging and treatment success. The logistic models demonstrated poor fit due to small sample size, were highly influenced by outliers in the data, and are therefore not presented. Receiver-operating-curve analysis was also limited by the small sample size, and a threshold absorbed dose could not established.

## DISCUSSION

Quantitative ^123^I-NaI imaging has shown great potential to assess whether further ^131^I-NaI treatment is warranted after attempted resensitization of RAI-refractory DTC with selumetinib. Significant inter- and intrapatient variability in the relative change in RAI uptake after selumetinib treatment was found in the present cohort. This variability suggests that, to fulfil the justification principle, patient-specific uptake changes should be assessed before RAI therapy proceeds. Furthermore, the RAI concentration within lesions before and after selumetinib treatment markedly differed between and within patients. No patient- or lesion-specific biomarkers were found to be predictive of uptake. Results of studies by Ho et al. suggest that a biomarker-directed strategy may be required, as redifferentiation using selumetinib in BRAF^V600E^-mutant patients appeared to be less successful (7*,*^19^).

The relative change in uptake did not correlate with baseline uptake, suggesting that selumetinib might also be effective in nonrefractory patients, who still have a degree of iodine uptake in lesions before starting treatment. Ho et al. (19) used selumetinib plus RAI therapy in a phase 3 randomized clinical trial on the adjuvant treatment of high-risk, resected DTC patients but could not show a statistically significant difference in complete remission when compared with RAI therapy alone. Further investigations are warranted on the use of selumetinib or other related drugs to improve the outcome in cohorts of advanced-DTC patients at risk of becoming RAI-refractory.

The wide range of RAI uptake observed is also reflected by the wide range of absorbed dose delivered to lesions from a fixed 5.5-GBq administration to patients with advanced DTC. This administration is considered standard practice in the United Kingdom and in line with national guidelines and was therefore used in the current study (20). The wide range agrees with results presented previously (21*,*^22^) and might explain the variations in outcome observed among patients. Personalized treatment planning based on the absorbed doses delivered could, therefore, potentially be warranted in this cohort.

The results suggest that personalized treatment planning using pretherapy ^123^I-NaI is feasible. Although the absolute accuracy decreases for absorbed doses higher than 50 Gy, pretherapy dosimetry correlated fairly well with posttreatment dosimetry. The average difference of −33% may be due to differences in imaging schedules, the possibility of a stunning effect of ^123^I-NaI or self-stunning of ^131^I-NaI (23*,*^24^*,*^25^), a delayed action of the selumetinib during the time before therapy, alterations of biokinetics due to prior administration of recombinant human thyroid-stimulating hormone (26), saturation of receptors, or the continued use of selumetinib before RAI therapy. The last of these possibilities has not yet been studied. To our knowledge, this is the first time that ^123^I-NaI pretherapy dosimetry has been shown to be indicative of the absorbed doses delivered from treatment in metastatic DTC patients. This result might have wide implications for molecular radiotherapy, by allowing for personalized treatment planning in combination with dosimetric methods to assess absorbed doses to organs at risk, such as whole-body and bone marrow dosimetry (27).

The large uncertainties in absorbed doses, brought about by the use of oversized volumes of interest, reflect the volume estimate uncertainties for small lesions and the significantly shorter half-life of ^123^I-NaI (28). Uncertainties are potentially smaller with ^124^I-NaI pretherapy dosimetry, which was not available for this study. The longer physical half-life of ^124^I-NaI than of ^123^I-NaI would allow for more accurate determination of the retention half-lives. ^123^I-NaI pretherapy dosimetry potentially overestimates the retention half-life (Supplemental Fig. 3) and, therefore, the absorbed dose delivered.

The present analysis has some limitations. The trial was not designed to have sufficient power to detect statistically significant effects on the dosimetry endpoints, and statistical testing did not account for multiple testing. The relatively small number of patients undergoing both pre- and posttherapy dosimetry is a limiting factor, and the statistical analysis should be considered exploratory. When feasible, future studies should be sufficiently powered to detect statistically significant effects and to identify key parameters affecting both the response to treatments before RAI therapy and the absorbed doses delivered. Additionally, since these types of data have a nested structure of lesions within patients, careful consideration should be given to the design of trials aiming to capture these data. Such a design will aid in avoiding or mitigating potential issues that may arise when using a multilevel modeling approach, such as the issues with model singularity encountered here. Follow-up analysis was limited by the short follow-up time, leading to inconclusive results with respect to the absorbed dose relationship due to a poor model fit. Response measurements of bone lesions using RECIST are considered difficult but can be performed for osteolytic lesions, the predominant type in thyroid cancer. Nevertheless, the lack of an absorbed dose relationship potentially reflects that 14 of the 39 lesions were found in bone. Similarly, an absorbed dose threshold could not be identified. Most absorbed doses delivered were estimated to be lower than the proposed absorbed dose thresholds for soft-tissue (29) and bone metastases (30), as is in line with the low overall response rate of 13%.

Absorbed dose–response relationships for advanced DTC (30) should be confirmed in multicenter clinical trials. Together with the results presented here, these future results would facilitate personalized treatment planning of RAI administrations.

## CONCLUSION

Quantitative SPECT/CT has shown large inter- and intrapatient variability in the effect of selumetinib on increasing the lesional RAI uptake in advanced RAI-refractory DTC. In addition, a large range of RAI uptake concentrations was observed in lesions at baseline. The absorbed doses delivered at therapy in this cohort can be estimated from a pretherapy dosimetry study. These findings suggest that future studies of redifferentiation therapy should use the combination of pretherapy quantitative imaging (to assess the effect of treatments to enhance RAI uptake) and dosimetry (to plan the activity of RAI administered to patients) to ensure that those patients achieving increased iodine uptake obtain maximum benefit from subsequent therapy. Our findings also likely have implications for the personalized treatment planning of patients with iodine-sensitive DTC.

## DISCLOSURE

Financial support was received from Cancer Research U.K. (CRUK) (CRUK reference CRUK/14/041), AstraZeneca U.K. Limited, the NIHR Biomedical Research Centre at RMH/ICR, and the National Institute for Health Research (NIHR). Sanofi Genzyme provided recombinant human thyroid-stimulating hormone. Sarah Brown, Glenn Flux, Jonathan Wadsley, Jan Taprogge, Iain Murray, Jonathan Gear, and Carla Abreu report funding or provision of study materials from CRUK, AstraZeneca, and Sanofi Genzyme during the conduct of the study. Jan Taprogge, Carla Abreu, Jonathan Gear, Iain Murray, and Glenn Flux report funding from the National Health Service to the NIHR Biomedical Research Centre at the Royal Marsden and the ICR and grants from Euratom research and training program 2014–2018 and the NIHR and NIHR Royal Marsden Clinical Research Facility. Jonathan Gear reports personal fees and honoraria from the European Association of Nuclear Medicine. Jonathan Wadsley reports personal fees and honoraria from Lilly, Eisai, Novartis, AAA, Bayer, Ipsen, and Roche. Amy Coulson reports grants and nonfinancial support from BMS/Celgene, Merck Sharpe & Dohme, Amgen, and Takeda. Sanofi Genzyme provided recombinant human thyroid-stimulating hormone. The research was developed with support from NCRI CTRad. NHS funding was provided to the NIHR Biomedical Research Centre at the Royal Marsden and the ICR. The RTTQA group is funded by the NIHR. We acknowledge infrastructure support from NIHR Royal Marsden Clinical Research Facility funding. This report is independent research funded by the NIHR. The views expressed in this publication are those of the authors and not necessarily those of the NHS, the NIHR, or the Department of Health and Social Care. No other potential conflict of interest relevant to this article was reported.

## Notes

KEY POINTS **QUESTION:** Can quantitative imaging and SPECT/CT-based lesional dosimetry be used to personalize RAI treatment for advanced thyroid cancer, which currently has a poor prognosis? **PERTINENT FINDINGS:** Pretherapy imaging was found to be a powerful tool to assist with the stratification of patients before further RAI therapy, and a strong positive correlation was found between pre- and posttherapy absorbed doses, indicating the suitability of pretherapy dosimetry for treatment planning. **IMPLICATIONS FOR PATIENT CARE:** Pretherapy quantitative imaging and dosimetry in RAI therapy for advanced thyroid cancer has the potential to inform treatment planning for individual patients and to alter patient management.
